# Impact of Pharmacists-Led Interventions in Primary Care for Adults with Type 2 Diabetes on HbA1c Levels: A Systematic Review and Meta-Analysis

**DOI:** 10.3390/ijerph19063156

**Published:** 2022-03-08

**Authors:** Claire Coutureau, Florian Slimano, Céline Mongaret, Lukshe Kanagaratnam

**Affiliations:** 1 Department of Research and Public Health, Reims University Hospital, 51092 Reims, France; lkanagaratnam@chu-reims.fr; 2 UR 3797 Vieillissement, Fragilité (VieFra), Faculty of Medicine, University of Reims Champagne-Ardenne, 51092 Reims, France; 3 Department of Pharmacy, Reims University Hospital, 51092 Reims, France; fslimano@chu-reims.fr (F.S.); cmongaret@chu-reims.fr (C.M.)

**Keywords:** diabetes mellitus, pharmacists, primary health care, meta-analysis

## Abstract

Type 2 diabetes mellitus (T2D) is responsible for an important premature mortality. Pharmacists involved in community-based pharmaceutical care services could help patients with diabetes through education and management as they participate in their regular and long-term care. This meta-analysis aimed to evaluate the association between interventions led by pharmacists in the primary care setting and mean change in HbA1c levels. Randomized controlled trials and quasi-experimental studies with a control group were included. Standardized mean differences (SMD) and their 95% confidence intervals (95% CI) were calculated to compare the mean change in HbA1c values between baseline and end of the intervention in each group. Subgroup analyses were performed to explore heterogeneity. Twelve articles were included. The results showed that pharmacist’s interventions significantly reduced HbA1c compared to usual care with an overall SMD of −0.67 (95% CI = [−0.87; −0.48], *p* < 0.0001). Even if no significant difference between subgroups were found, the reduction of HbA1c seemed more important when baseline HbA1c was ≥8.5%, the intervention occurred monthly, in a primary care center and in countries with a lower human development index. Our results suggest that pharmacists-led interventions in the primary care setting can improve glycemic control for adults with T2D.

## 1. Introduction

Diabetes mellitus is a chronic disease that affects an increasing number of people around the world. According to a World Health Organization (WHO) report, its age-standardized prevalence worldwide skyrocketed from 4.7% of the adult population in 1980 to 8.5% in 2014, hence, representing 422 million people [1].

The most frequent type of diabetes mellitus, representing 85% to 95% of diabetes, is type 2 diabetes mellitus (T2D). The main risk factors for T2D are familial history of diabetes, sedentary lifestyle and obesity [2].

The global economic burden of diabetes constantly increases and it is estimated that it would exceed two trillion USD in 2030 [3].

Above all, diabetes mellitus is responsible for a staggering premature mortality and leads to early morbidity. Guidelines have historically selected glycemic control as the main outcome measure for diabetes mellitus care. Glycated hemoglobin (HbA1c) reflects the mean blood sugar level for the last three months and the American Diabetes Association (ADA) recommends its measurement at least twice a year [4]. Hyperglycemia is significantly associated with diabetes risks for complication especially microvascular. Stratton et al. have highlighted that for each 1% decrease in mean HbA1c, the decrease of the risk of complications from diabetes is of 21% [5]. HbA1c is thus considered a good marker for the risk of complications among population with diabetes.

Diabetes mellitus leads to and is associated with cardiovascular diseases. Co-existence of essential hypertension and T2D is very common and hypertension is twice more common in patients with diabetes than in those without diabetes [6]. Both diseases are at risk of cardiovascular events and lowering blood pressure is particularly beneficial in patients with diabetes. Thus, diabetes care should include hypertension management.

Patients are in frequent contact with pharmacists, making the latter particularly appropriate healthcare workers for supporting people with long-term conditions like diabetes mellitus [7].

Moving from a drug retail role to healthcare providers, they are involved in the regular and long-term care of patients with diabetes [8]. They also play a key role in acute complication identification, referring patients to their general practitioner if needed. An educational role is also awaited from the pharmacist to address underlying self-determinants or barriers that may be negatively impacting an individual’s ability to manage their condition in a broader sense. Finally, some countries have extended pharmacists’ skills to monitoring, like community pharmacists in Quebec (Canada) who are now allowed to prescribe glycemia and HbA1c measurements.

A cohort study, part of the Asheville project in the USA in the beginning of the 2000s, showed a better glycemic control at short- and long-term with targeted and repeated interventions in direct contact with the patient, by trained community pharmacists [9].

Clinical trials and meta-analyses have also highlighted a beneficial effect of various interventions led by pharmacists for patients with diabetes. Indeed, a meta-analysis by Presley et al., focusing on pharmacist’s interventions to improve medication adherence, showed beneficial effects of these interventions on several outcomes including HbA1c for adult patients with type 1 or type 2 diabetes [10]. Pharmacists’ interventions could therefore lead to a better diabetes control and a reduction of the risk of complication.

Nonetheless, to our knowledge, none of the meta-analyses showing a beneficial effect of pharmacists interventions on diabetes control [10,11,12,13,14,15,16,17,18] focused on interventions in the primary care setting only (community pharmacies or primary care centers) in the control of T2D. Firstly, other meta-analyses on the same topic included studies with type 1 and type 2 adults with diabetes, without distinction between these two types [10,11,12,13,18]. Type 1 diabetes mellitus (T1D) is different from T2D regarding origin, patient profile, risk factors, and treatment. Patients with T1D are generally younger and educational approaches to manage insulin therapy, glycemic monitoring, and very long-term conditions without curative perspectives are a priority. The care of patients with T2D is different and includes several pharmacological treatment approaches starting with oral antidiabetic drugs. Patients’ profiles are different from patients with T1D in terms of comorbidities management, long-term conditions, and challenges for morbi-mortality reduction. Consequently, pharmacists’ activities and interventions are different between these patients. Pharmacists’ activities proposed to patients with T2D have specific outcomes including the promotion of physical activity, healthy diet, and lifestyle. Secondly, most published meta-analyses reported studies led in different care settings, including tertiary care/hospital setting [10,11,13,14,15,16,17,18]. Regarding settings, primary care represents the cornerstone of the health system and the first contact for initial and long-term diabetes management. Most subjects with T2D see their general practitioner and pharmacist several times a year. Indeed, primary care providers deliver the majority of care to patients with T2D [19].

To our knowledge, no meta-analysis, showing the significant impact of pharmacists’ interventions on HbA1c, were focused on interventions in the primary care setting only (community pharmacies or primary care centers) in the control of T2D.

Thus, the primary objective of this systematic review and meta-analysis is to evaluate the association between interventions led by community pharmacists or pharmacists in primary care centers and the mean change in HbA1c levels. The secondary objective is to study the association between these interventions and blood pressure control.

## 2. Materials and Methods

### 2.1. Search Strategy

A bibliographic search was performed in the following databases: Pubmed, Cochrane, and Embase. The two keywords “diabetes” and “pharmacist” were used, combined using the Boolean Operator “AND”. All studies were searched from the start date of each database, up to July 2021. The detailed search strategy is given in Appendix A. In every meta-analysis identified about pharmacists’ interventions and T2D, references were checked for potential additional studies

### 2.2. Study Selection

The study selection was performed by two reviewers (C.C. and L.K.). First, they reviewed independently all titles and abstracts. Any disagreement was discussed and then resolved among the two reviewers (C.C. and L.K.) and when necessary the opinion of 2 other reviewers was requested (C.M. and F.S.). Then, all full texts of the articles included based on the titles and abstracts were independently reviewed by the same two persons (C.C. and L.K.). Any disagreement on the full text selection was discussed and solved, with 2 other reviewers if necessary (C.M. and F.S.).

#### 2.2.1. Inclusion Criteria

Randomized controlled trials and quasi-experimental studies with a control group were included if they evaluated an intervention performed by a pharmacist in the primary care setting (community pharmacies or primary care centers) for patients with T2D, compared with usual pharmaceutical care. To be included, each article should have reported mean HbA1c levels in each group at baseline and at the end of the study (with standard deviation), or the mean change in HbA1c levels between baseline and the end of the study in each group (with the standard deviation of this mean difference or its 95% confidence interval (95% CI)).

#### 2.2.2. Exclusion Criteria

Studies were excluded if

- they were retrospective studies, observational studies and quasi-experimental pretest posttest designs with no control group;
- the intervention was not considered to be part of the primary care (e.g., hospital setting, clinical pharmacist interventions);
- there was no physical encounter between the pharmacist and the patient or if no education or counselling was proposed by the pharmacist;
- the evaluated intervention was performed by a multidisciplinary team (e.g., pharmacists associated with nurses, dieticians, endocrinologist) because it would have been difficult to estimate the precise impact of the pharmacist, except for the cooperation between the pharmacist and the general practitioner because it constitutes the cornerstone of primary care and the pharmacist often makes recommendations to the general practitioner;
- they evaluated the impact of the intervention on other chronic diseases (e.g., arterial hypertension) with no distinct results for the patients with diabetes.

### 2.3. Outcomes

The primary outcome of this meta-analysis was the mean change in HbA1c levels, commonly used in clinical practice to monitor glycemic control.

The secondary outcome was the mean change in systolic blood pressure (SBP). Focus was made on SBP only, as SBP and diastolic blood pressure (DBP) frequently display a linear relationship [20] and epidemiological studies suggest that SBP should be the primary target of antihypertensive therapy [21].

To evaluate the association between the pharmacist’s intervention and the outcomes, the mean change in HbA1c and SBP between baseline and the end of the intervention in each group was used.

### 2.4. Data Extraction

One of the reviewers (C.C.) extracted the data from the included studies. All data were double-checked by the second reviewer (L. K.). The extracted information from the full text of the study were: first author, year of publication, study design, country of the study site, intervention setting (i.e., community pharmacies or primary care centers), intervention type (e.g., education, medication review), intervention duration and frequency, pharmacist’s specific training for the study, inclusion and exclusion criteria of the patients, patients’ mean age (years), number of subjects in each group, mean baseline HbA1c (%), mean baseline SBP (mmHg), mean final HbA1c and/or mean change between baseline and the end of the intervention (and standard deviation or 95% CI), mean final SBP and/or mean change between baseline and the end of the intervention (and standard deviation or 95% CI). We recorded the level of development of each study’s country using the Human Development Index (HDI) [22]. The HDI is calculated based on 3 key elements of human development: life expectancy at birth, education and national income per capita. A HDI of 0.8 or higher indicates a very high human development of the country.

### 2.5. Risk of Bias Assessment

To assess the risk of bias of the included studies, the Cochrane’s risk of bias criteria for Effective Practice and Organization of Care (EPOC) reviews was used [23]. This tool can be used for randomized trials, non-randomized trials and controlled before-after studies that evaluate the effects of health care interventions. Nine criteria were investigated: random sequence generation, allocation concealment, protection against contamination, selective outcome reporting, knowledge of the allocated interventions adequately prevented during the study, incomplete outcome data, baseline outcome measurements similar (i.e., baseline HbA1c levels), baseline characteristics similar, and other risks of bias.

Each item was scored as “Low risk”, “High Risk”, or “Unclear Risk”. Two reviewers (C.C. and L.K.) completed the assessment independently and any disagreement was discussed and solved. The EPOC risk of bias tool is presented in Supplementary Materials file S1.

Moreover, potential publication bias was evaluated with a funnel plot.

### 2.6. Statistical Analysis

The association between each outcome (HbA1c and SBP) and the intervention was evaluated quantitatively by meta-analysis, using random-effect models (DerSimonian-Laird method). Inverse variance weighting was used for pooling, that is, the weight given to each study was the inverse of the variance of the effect estimate. Pooled effect estimates were calculated as a weighted average of the intervention effects estimated in the individual studies.

For the primary outcome, the mean change in HbA1c levels between the baseline and the end of the intervention was compared between the intervention and the control group. Standardized mean differences (SMD) were calculated using the Hedges’ g statistic and presented with their 95% CI.

When mean difference between baseline and the end of the intervention in each group was missing, it was calculated from mean baseline and mean final HbA1c in each group and when the standard deviation of the mean difference was missing, it was computed from the 95% CI of the mean difference. If it was not possible to compute the missing values, especially regarding the standard deviation of the mean difference before and after intervention, the corresponding author was contacted and asked for the missing data. Between-study heterogeneity was assessed using the Q-test, the I^2^ statistic (percentage of between-study variation due to statistical heterogeneity rather than chance alone), and the τ^2^ (between-study variance, using the DerSimonian–Laird estimator).

For the secondary outcome (SBP), the same analysis as for HbA1c was conducted.

Subgroup analyses according to baseline HbA1c levels, age of the included participants, duration of intervention, frequency of intervention, country development (HDI) and intervention setting (community pharmacies or primary healthcare centers/clinics/units) were performed to explore heterogeneity among studies on the primary outcome, as recommended by Cochrane [24]. Selection of variables for subgroups analyses was done from previous studies and meta-analyses about diabetes and pharmacist’s interventions [17].

The characteristics of each group were described with the number of included patients, the mean age of the included patients, the mean baseline HbA1c levels, and the mean baseline SBP.

A sensitivity analysis was performed with studies for which mean HbA1c levels at the end of the intervention (and their standard deviation) were available to assess the robustness of the primary analysis results.

All analyses were performed with R software, version 4.0.5 (R Core Team (2020). R Foundation for Statistical Computing, Vienna, Austria), using the meta package (version 5.2-0, Balduzzi S, Rücker G, Schwarzer G, 2019).

This meta-analysis was performed in accordance with the Preferred Reporting Items for Systematic Reviews and Meta-analysis (PRISMA) recommendations [25] (Supplementary Materials file S2).

## 3. Results

**Figure 1:**
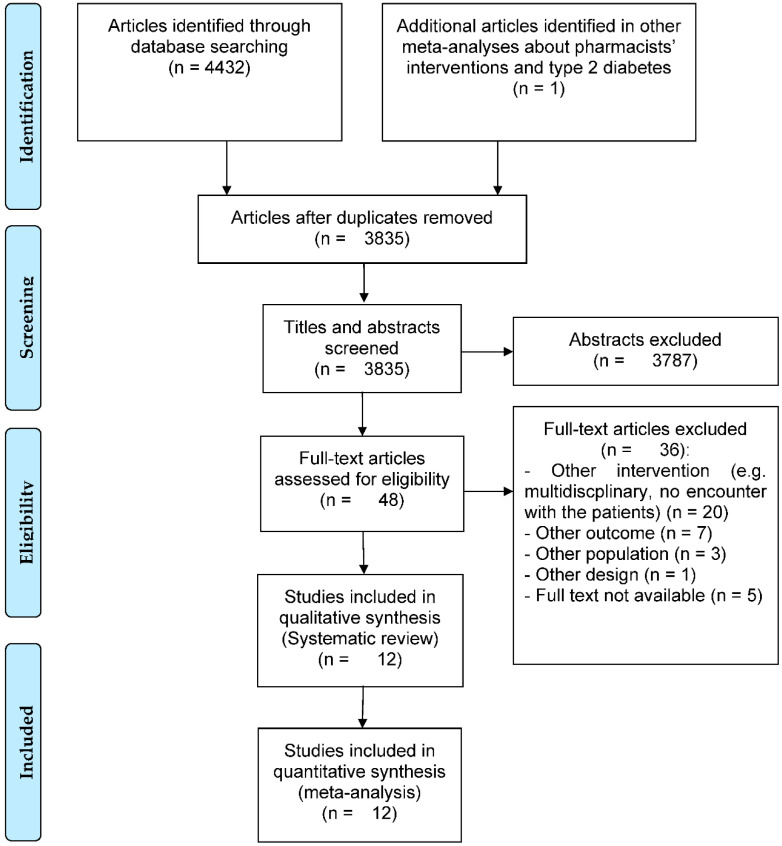
Flow chart of study selection.

Through database and reference searching, a total of 3835 abstracts were screened and 48 full-text articles were assessed for eligibility. At the end of the full-text assessment, 12 articles [26,27,28,29,30,31,32,33,34,35,36,37] were included in this meta-analysis. The main reasons for exclusion were interventions not corresponding to the inclusion criteria (e.g., secondary or tertiary care, no education or counselling), no data on HbA1c in each group and no full-text found (e.g., conference abstract). The details are shown in Figure 1.

### 3.1. Study Characteristics

The 12 studies included in this review were conducted in different countries all over the world: four in Europe [26,31,35,36] (i.e., United Kingdom, Spain, Belgium, and France), one in Australia [34], one in the United States of America [29] (USA), two in Brazil [28,37], and four in the Middle East and Asia [27,30,32,33] (i.e., Malaysia, Indonesia, Iran, Pakistan). Eleven studies were randomized controlled trials [26,27,29,30,31,32,33,34,35,36,37] and one study was a quasi-experimental controlled study [28]. Most of the studies took place in community pharmacies [26,28,29,31,32,34,35,36] (eight studies), the others in primary care units/clinics/centers [27,30,33,37]. The duration of the pharmacist intervention ranged from 5 to 13 months. The majority of these interventions occurred monthly [27,28,30,31,32,34,35,37].

All evaluated interventions provided education about diabetes to the patients, the topics varied according to the study (e.g., lifestyle, self-care, diet, adherence, complications of diabetes, drug use). Most pharmacists also provided medication review [26,27,28,29,31,32,33,34,37] (nine studies) and some, who identified drug related problems, formulated pharmacist’s interventions to the prescribers [27,28,29,33,37]. In 11 studies, the pharmacists followed a specific training program about diabetes [26,27,28,29,30,31,32,34,35,36,37].

The population of interest was slightly different among the studies with different inclusion and exclusion criteria, especially regarding the age, the HbA1c level and the use of insulin. The detailed characteristics of each study are presented in Table 1.

The overall mean age of the study participants ranged from 50.4 to 66.6 years old. The overall mean baseline HbA1c level ranged from 7.5% to 10.9% and overall mean baseline SBP ranged from 119.0 to 146.7 mmHg (Table 2).

### 3.2. Risk of Bias Assessment

The risk of bias assessment with the EPOC criteria showed variability in the quality of the included studies. There was low risk of bias induced by the knowledge of the allocated intervention and low risk of bias of selective outcome reporting. Regarding the random sequence generation, 11 studies were randomized controlled studies but four of them did not describe with enough details the randomization process (unclear risk) and one study was a non-randomized controlled one (high risk). There were some concerns regarding differences between the two groups of the baseline characteristics (high or unclear risk for six studies) and the baseline outcomes (high risk for two studies). The two most important risks of bias, found at high risk in 7 out of 12 studies, were the presence of incomplete outcome data for patients at the end of the intervention regarding the primary outcome (e.g., patients lost to follow-up) and the risk of contamination of the intervention between the groups when the randomization was at the patient level and not at the pharmacy or center level. The summary of these results is available in Appendix B.

Regarding publication bias, the analysis of the funnel plot did not find any major bias (Appendix C).

### 3.3. Meta-Analysis

#### 3.3.1. Primary Outcome

Of the 12 studies included in this meta-analysis, 10 reported adequate data to be included in the primary analysis [26,27,28,29,30,31,32,34,35,37] (i.e., mean change in HbA1c values between baseline and the end of the intervention and the standard deviation of this difference in each group were available or necessary data to compute them were available). Indeed, it was not possible for two studies to calculate, or collect the missing data regarding the standard deviation of the mean difference before and after intervention [33,36].

**Figure 2:**
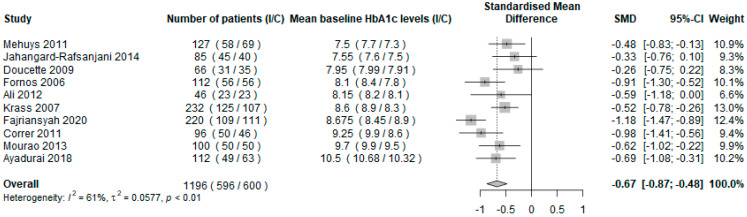
Forest plot of the mean difference in HbA1c levels in the intervention group compared with the usual pharmaceutical care group using random effects model. Abbreviations: I = intervention group; C = control group; HbA1c = glycated haemoglobin.

The results of the primary analysis, evaluating the impact of the pharmacist intervention using the mean change in HbA1c values between baseline and the end of the intervention in the intervention group compared to the usual care group, are presented in Figure 2. A total of 1196 subjects were included, and seven studies showed a beneficial effect [27,28,30,31,34,35,37]. The overall SMD was −0.67 (95% CI = [−0.87; −0.48], *p* < 0.0001), showing that pharmacist’s interventions significantly reduced HbA1c compared to usual care in T2D patients. Heterogeneity among the included studies was high and significant (I^2^ = 61%, *p* < 0.01).

The sensitivity analysis, comparing the mean HbA1c level at the end of the intervention in the intervention group compared to the usual care group, included nine studies [26,27,30,31,32,33,34,35,36] and 1391 patients, which had the necessary data available (i.e., mean HbA1c values and standard deviation at the end of the intervention, in each group). This analysis showed a significant reduction of HbA1c in the intervention group compared to the usual care group (SMD = −0.58 95% IC = [−0.93; −0.22], *p* = 0.0016). This result is consistent with the primary analysis.

#### 3.3.2. Subgroups Analysis

No significant difference, in the reduction of HbA1c levels, between subgroups created according to baseline HbA1c level (<8.5% vs. ≥8.5%), age (≤60 years old or >60 years old), duration of intervention (≤6 months vs. >6 months), frequency of intervention (every month or less often than monthly), HDI (≥0.8, indicating a very high human development [38] vs. <0.8), and intervention setting were found.

**Figure 3:**
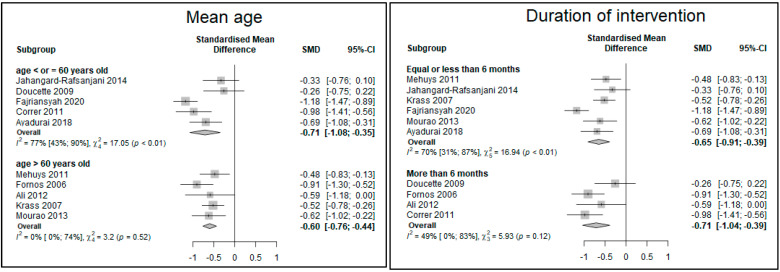

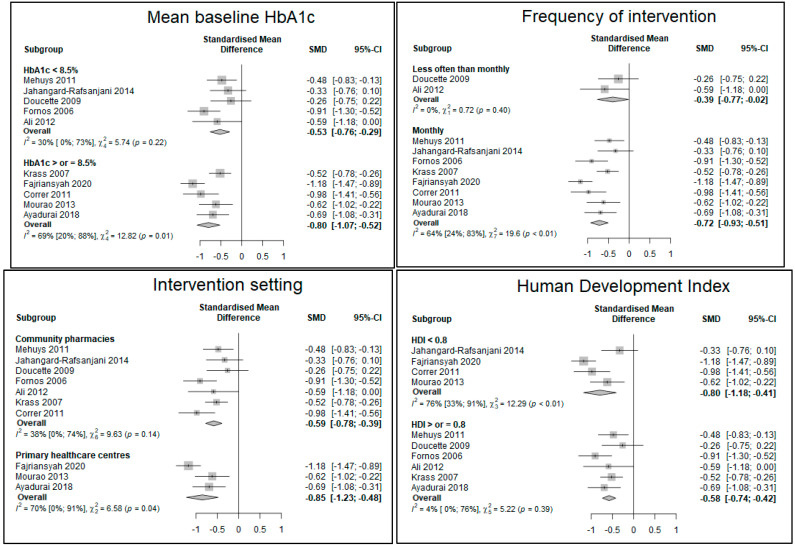
Forest plot of the subgroups analyses of the mean difference in HbA1c levels in the intervention group compared with the usual pharmaceutical care group using random effects model. Abbreviations: I = intervention group; C = control group; HbA1c = glycated hemoglobin; HDI = Human Development Index.

The reduction of HbA1c seemed more important when baseline HbA1c was ≥8.5%, the intervention occurred every month, in a primary care center and in countries with a lower human development index (Figure 3).

#### 3.3.3. Secondary Outcome

Five studies, representing 565 patients, reported adequate data to be included in the analysis of the secondary outcome [27,28,29,34,37]. The impact of the intervention on SBP was evaluated using the mean change between baseline and final measurements in the intervention group compared to the control group.

**Figure 4:**
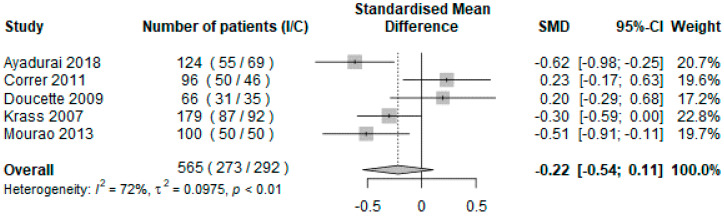
Forest plot of the mean difference in systolic blood pressure in the intervention group compared with the usual pharmaceutical care group using random effects model. Abbreviations: I = intervention group; C = control group; HbA1c = glycated hemoglobin.

The results were not significant, with an overall SMD of −0.22 (IC 95% = [−0.54; 0.11], *p* = 0.19). Heterogeneity was high and significant (I^2^ = 72%, *p* < 0.01) (Figure 4).

## 4. Discussion

The results highlight the beneficial effect of pharmacist-led interventions in the primary care setting for T2D patients on glycemic control as measured by HbA1c.

These results are consistent with other meta-analyses on this topic when including T1D and T2D patients [12] or studies led in the secondary and tertiary care setting [11].

The two most important points of the pharmacist’s interventions in the included studies were education about diabetes mellitus and medication review.

When accurately described in the articles reviewed, most of the interventions in the included studies referred to the diabetes self-management education (DSME). The ADA defines DSME as an education, brought by all health care professionals, to improve knowledge and aptitudes of the patient to perform diabetes self-care [39]. Seven primordial behaviors for self-management of diabetes have been defined because evidence showed that they were associated with better outcomes [40]. Among these identified behaviors, one can find healthy diet, physical activity, adherence to medication and monitoring of blood glucose. These key elements are reported in the studies of this analysis. It is crucial that this education is part of each professional care for patients with T2D.

A closer look at the interventions on diabetes treatments pointed out that 10 of 12 studies included drug review/management. Medication review consists in a structured evaluation of a patient’s medicines with the aim of optimizing medicines use and improving health outcomes. This entails detecting drug related problems and formulating interventions [41]. A review from Jokanovic et al. in 2017, about pharmacist-led medication review, reported 35 out of 45 studies with beneficial effects on glycemic control [42].

Even if the comparisons between subgroups were not statistically significant, the pharmacist intervention seemed to have more beneficial effects on HbA1c levels in patients with higher baseline HbA1c values, living in countries with a lower HDI, when it was conducted every month and in a primary care center. These results are consistent with another meta-analysis led by Aguiar et al. which highlighted that the impact of the pharmacist tended to be more important when the mean baseline HbA1c levels were more than 9.0% and when the intervention was led in other outpatient settings compared with a community pharmacy [17].

These findings regarding the setting could be explained by the fact that primary care centers often offer to the patients a multidisciplinary care and direct cooperation between professionals, with focus on T2D for specialized centers. These could have influenced the intervention and led to better results in these patients, compared to the community pharmacies where it can be more difficult for the pharmacists to have interactions with other professionals [43,44]. Moreover, there might be a different level of experience in the management of chronic diseases between pharmacists working in different settings. Nonetheless, the role of the community pharmacist is crucial in diabetes care, as the most accessible healthcare professional in many countries, and their full integration in existing care models of chronic diseases needs to be strengthened [7].

Concerning the benefits on higher baseline HbA1c levels and in developing countries, the results are consistent with literature data [11,14]. Indeed, diabetes prevalence increases quickly in developing countries while access to the necessary care can be challenging [45]. Patients with poorer glycemic control might lack adequate care and education that can be highly improved with pharmacists’ interventions. Margins for improvement are also more important with higher baseline HbA1c levels. It has already been discussed that there is a critical need for efficient primary care structures to manage chronic diseases in low and middle-income countries [46].

Despite including only controlled trials led in the primary care setting, heterogeneity was high. Statistical heterogeneity refers to the differences between studies in the effect of the evaluated intervention that are not attributable to chance alone. It can be explained, among many other elements, by the study setting, the characteristics of the study population and the type of intervention [47].

This heterogeneity was explored with subgroups analyses. Indeed, it is interesting to note that heterogeneity was less important (<50%) in studies where overall mean baseline HbA1C was less than 8.5%, age was above 60 years old, the intervention lasted more than six months, the HDI of the country was more than 0.8 and the setting was a community pharmacy.

Heterogeneity can also be explained by methodological differences between studies. One of the biggest risks of bias was the risk of contamination of the intervention. It is the case when randomization is not at the pharmacy or center level. Indeed, a professional who has been trained for a specific intervention is at risk of also modifying their usual care practice for patients in the control group. These patients may also interact with patients in the intervention group, benefit from advice and change their self-care attitude. This can dilute the effect of the intervention and lead a real difference between groups to go unnoticed. Among the 10 studies included in the primary analysis, three brought non-significant results [26,29,32] and they were all RCTs with randomization at the patient level. However, contamination when randomization is at the patient level can be avoided with a good study design. Cluster-randomized trials can be difficult to implement because they need a larger sample size and can be associated with recruitment bias [48].

Regarding the secondary outcome, unlike a meta-analysis performed by Fazel et al. [13], no beneficial effect of the pharmacist on SBP was found. This result might be explained by a lack of power, having only 565 patients included, and by the fact that the interventions were really focused on diabetes care, and not especially on blood pressure control, even if this is an important element of care in this population with a higher cardiovascular risk.

This meta-analysis was, to our knowledge, the first one to focus especially on pharmacist-led interventions in the primary care setting for patients with T2 diabetes. Included studies were only controlled trials, owing to their high level of evidence. In order to be exhaustive and prevent missing potential studies, broad research keywords were used in each database.

However, this study has some limitations. The main one might be the small number of included studies. Precise inclusion criteria were used to focus only on T2D and primary care but this allowed only 12 studies to be included, out of the 3835 abstracts initially screened. Moreover, it was not possible to retrieve the necessary data for the primary analysis for 2 studies. But the sensitivity analysis, performed with studies for which mean HbA1c levels at the end of the intervention was available, included these two studies and brought similar results. The lack of statistical power might explain the non-significant results between subgroups. Moreover, some of the variables chosen for subgroups analyses might be associated. It would have been interesting to explore the effect of each variable independently of the others on the reduction of HbA1c level with a meta-regression but the number of included studies in this meta-analysis was not sufficient.

## 5. Conclusions

The results of this systematic review and meta-analysis show that pharmacists-led interventions in the primary care setting can improve glycemic control for adults with type 2 diabetes. Long-term and repeated interventions, including thorough patient education about the disease, prevention of complications and medication review can lead to a significant improvement of diabetes control. HbA1c is a biomarker reflecting glycemic control and remains an intermediate endpoint in the context of T2D evolution and complications. Further studies are needed to evaluate the impact of these interventions performed by pharmacists on clinical outcomes, including microvascular but also macrovascular complications such as cardio-vascular events.

## Figures and Tables

**Table 1:** Study characteristics.

Study	Country; HDI	Study Design	Intervention Duration	Setting	Intervention Type	Frequency	Pharmacist Training	Inclusion/Exclusion Criteria
Ali 2012 [26]	United-Kingdom; HDI = 0.932	Randomized controlled study; Randomization at the patient level	12 months	2 community pharmacies	Pharmaceutical care package: targeted medicine use review (compliance and counseling), comprehensive and individualized education, lifestyle modification counseling	Every month the first 2 months, then every 3 months	8 h training program: update on diabetes management and referrals, overview of the use of diagnostic equipment	T2D, >18 y/o, oral medication (no insulin), no significant co-morbidity, HbA1C ≥ 7%
Ayadurai 2018 [27]	Malaysia; HDI = 0.810	Randomized controlled study; Randomization at the patient level	6 months	7 primary healthcare practices (health clinics)	Simplified tool to manage T2D: medication related concerns, recommendations to the prescribers, education	Monthly	2h online training program to use the tool	T2D, >21 y/o, on multiple medications (including for other chronic conditions) and/or have other diseases in addition to diabetes, HbA1C > 8% (or fasting blood sugar > 7.0 mmol/L or 2 h post prandial sugar level > 8.5 mmol/L)
Correr 2011 [28]	Brazil; HDI = 0.765	Quasi-experimental controlled study	12 months	6 community pharmacies	PFU program: comprehensive and systematic medication outcome assessment, suggesting changes in the medication, education	Monthly	Training on basic concepts and procedures of pharmacotherapy follow-up, diabetes care, glucose and blood pressure measurement	T2D, >30 y/o, using either oral hypoglycemiants or insulin
Doucette 2009 [29]	USA; HDI = 0.926	Randomized controlled study; Randomization at the patient level	12 months	7 community pharmacies	Assessment of clinical markers, review of medications and self-care behaviors, identifying drug therapy problems, recommendation of drug therapy change and education (diabetes self-care)	Quarterly	Training in diabetes management: 15 h self-study certificate program in diabetes management and live training (pathophysiology, therapeutics, self-care…)	T2D, HbA1C ≥ 7.0%
Fajriansyah 2020 [30]	Indonesia; HDI = 0.718	Randomized controlled trial; Randomization at the center level	6 months	4 primary health care centers (Puskesmas)	Education about T2D causes and symptoms, importance of therapy, therapies available, guidelines for the treatment, purpose of controlling blood sugar levels, lifestyle	Monthly	8 h training with experts	T2D, 18 ≤ age ≥ 65 y/o, HbA1C ≥ 6.5%
Fornos 2006 [31]	Spain; HDI = 0.904	Randomized controlled trial; Randomization at the patient level	13 months	14 community pharmacies	PFU program: prevent, detect and solve the problems related to the drugs used, information about drug (correct use, adverse reaction, interaction), assessment of lifestyle and health education actions	Monthly	Educational program to increase knowledge about diabetes and 18h of trainingin the PFU program and in the proper use of the measuring tools	T2D, on oral antidiabetics > 2 months
Jahangard-Rafsanjani 2014 [32}	Iran; HDI = 0.783	Randomized controlled trial; Randomization at the patient level	5 months	1 community pharmacy	Diabetes education program on diet management, physical activity, diabetes complications, discussion about medication-related problems and self-care issues	Monthly	4 h training: pathophysiology and pharmacotherapy, 3-day workshop on diabetes education	T2D, oral hypoglycemic medications, HbA1C > 7% within the preceding month
Javaid 2019 [33]	Pakistan; HDI = 0.557	Randomized controlled trial; Randomization at the patient level	9 months	1 primary care clinic	Comprehensive pharmaceutical care plan: assessment for drug related problems, suggestions for therapy changes, verbal and readable education (insulin administration, medication adherence, treatment goals, self-care, dietary, lifestyle, monitoring of blood glucose, footcare and hygiene...)	Quarterly	NA	T2D, >18 y/o, HbA1c > 8%,
Krass 2007 [34]	Australia; HDI = 0.944	Randomized controlled trial; Randomization at the pharmacy level	6 months	56 community pharmacies	Review of self-monitoring of blood glucose, disease, medication, self-management and lifestyle education (physical activity, weight loss), adherence support, medication review and detection of drug-related problems	Monthly	Diabetes education manual for self-directed learning and a 2-day workshop (pharmacotherapy, dietary management, role-playing exercises, training on the use of measuring tools)	T2D with: HbA1c ≥ 7.5%, with ≥ 1 oral glucose lowering medication or insulin; HbA1c ≥ 7.0%, ≥ 1 oral glucose lowering medication or insulin AND ≥ 1 anti-hypertensive, angina or lipid-lowering drug
Mehuys 2011 [35]	Belgium; HDI = 0.931	Randomized controlled trial; Randomization at the pharmacy level	6 months	66 community pharmacies	Education on diabetes and its complications, about the correct use of oral hypoglycemic agents, facilitation of medication adherence, healthy lifestyle education, reminders about annual eye and foot examinations	At each prescription-refill visit	Training session on the pathophysiology of diabetes and its non-pharmacological and pharmacological management	T2D, 45 ≤ age ≥ 75, BMI ≥ 25 kg/m^2^, treatment with oral hypoglycemic medication for ≥ 12 months
Michiels 2019 [36]	France; HDI = 0.901	Randomized controlled trial; Randomization at the pharmacy level	6 months	174 community pharmacies	Structured and tailored information on diabetes diet, medication management and diabetes complications	3 interviews	Information on the study by phone, face to face training and a guide explaining how to perform the interviews	T2D, HbA1c level > 7%, with ≤3 different oral antidiabetic drugs
Mourao 2013 [37]	Brazil; HDI = 0.765	Randomized controlled trial; Randomization at the patient level	6 months	6 primary health care units	Care plan including pharmacotherapy changes if necessary and education about non-pharmacological issues (aetiology, pathophysiology, complications, treatment goals, lifestyle) and pharmacological treatments (proper dosage, side-effects, drug storage)	Monthly	Training in pharmaceutical care and diabetes management	T2D, ≥18 y/o, with post-prandial capillary glucose ≥180 mg/dL and HbA1c ≥ 7 %, under ≥1 oral antidiabetic medications for ≥6 months

Abbreviations: HDI = Human Development Index; h = hour; T2D = type 2 diabetes mellitus; y/o = years old; HbA1c = glycated hemoglobin; PFU = Pharmacotherapy follow-up; NA = not available.

**Table 2:** Patients characteristics.

Study	Patients (n)		Mean Age (years)		Mean Baseline HbA1c (%)		Mean Baseline SBP (mmHg)	
	Intervention	Control	Intervention	Control	Intervention	Control	Intervention	Control
Ali 2012 [26]	23	23	66.4	66.8	8.2	8.1	146.3	136.2
Ayadurai 2018 [27]	55	69	55	58	10.68	10.32	137	137.8
Correr 2011 [28]	50	46	58.1	59.5	9.9	8.6	135	147.7
Doucette 2009 [29]	31	35	58.7	61.2	7.99	7.91	118.2	119.8
Fajriansyah 2020 [30]	109	111	mean age of both groups: 57.7		8.45	8.9		
Fornos 2006 [31]	56	56	62.4	64.9	8.4	7.8	143	148
Jahangard-Rafsanjani 2014 [32]	45	40	57.3	55.9	7.6	7.5	132	136.4
Javaid 2019 [33]	83	52	50.3	50.4	11	10.7	145	133
Krass 2007 [34]	157	142	mean age of both groups: 62		8.9	8.3	135	133
Mehuys 2011 [35]	148	132	63	62.3	7.7	7.3		
Michiels 2019 [36]	189	188	65.1	66.3	7.9	7.7	134.4	137
Mourao 2013 [37]	50	50	60	61.3	9.9	9.5	152.9	140.4

Abbreviations: HbA1c = glycated hemoglobin; SBP = systolic blood pressure.

**Figure A1:**
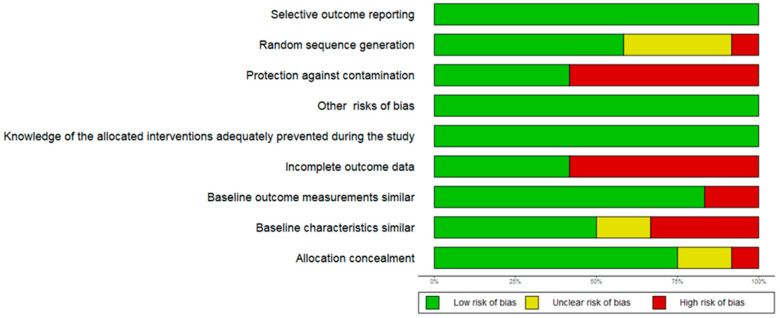
B1 Summarized risk of bias of the included studies.

**Figure A2:**
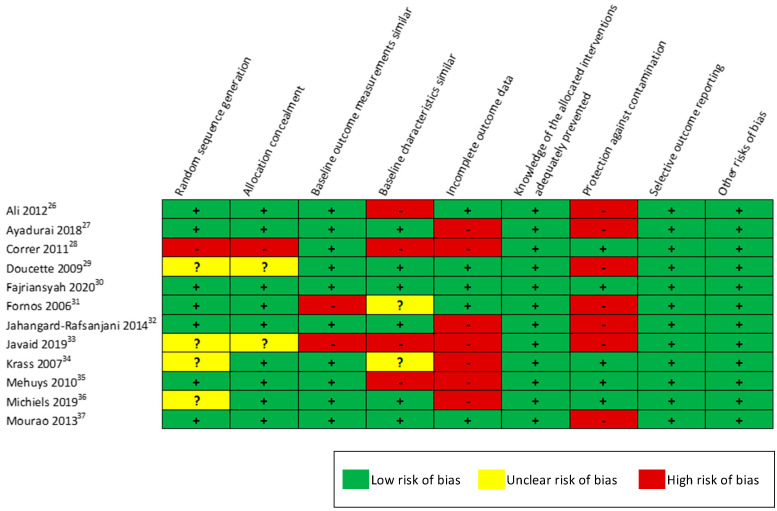
B2 Detailed risk of bias of the included studies.

**Figure A3:**
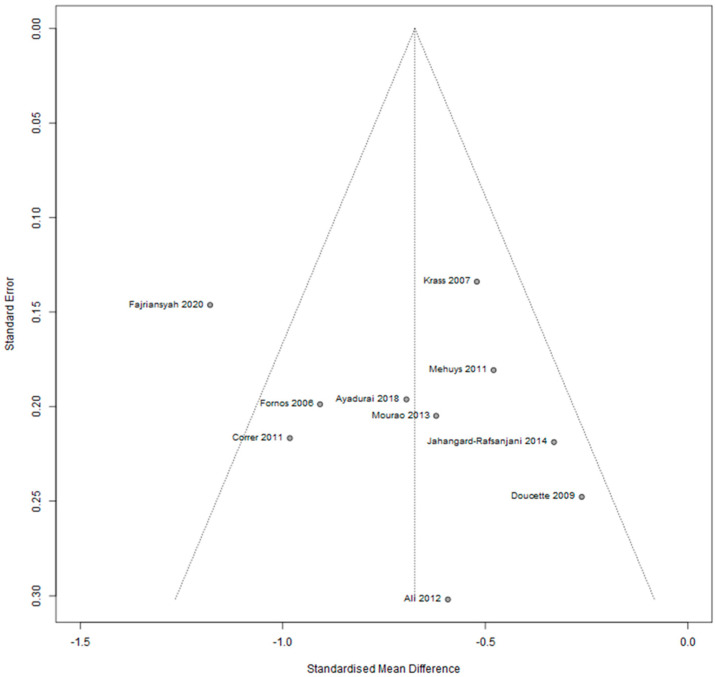
Funnel plot.

## Supplementary Materials

The following supporting information can be downloaded at: https://www.mdpi.com/article/10.3390/ijerph19063156/s1, Document S1: Suggested risk of bias criteria for EPOC reviews; Table S2: PRISMA 2009 Checklist.

## Appendix A

Search Strategy

PubMed (July 5th, 2021): (“diabetes mellitus”[MeSH Terms] OR (“diabetes”[All Fields] AND “mellitus”[All Fields]) OR “diabetes mellitus”[All Fields] OR “diabetes”[All Fields] OR “diabetes insipidus”[MeSH Terms] OR (“diabetes”[All Fields] AND “insipidus”[All Fields]) OR “diabetes insipidus”[All Fields]) AND (“pharmacists”[MeSH Terms] OR “pharmacists”[All Fields] OR “pharmacist”[All Fields])

Cochrane (July 5th, 2021): (Trials matching “diabetes” in All Text AND “pharmacist” in All Text)—(Word variations have been searched)

Embase (July 6th, 2021): Pharmacist:ab,ti AND diabetes:ab,ti AND [embase]/lim NOT ([embase]/lim AND [medline]/lim)
